# Increasing rural nurses’ awareness of a statewide health information resource: an educational outreach initiative

**DOI:** 10.5195/jmla.2019.542

**Published:** 2019-04-01

**Authors:** Kathryn Vela, Tania Bardyn

**Affiliations:** Health Sciences Librarian, Washington State University, Spokane, WA 99210, kathryn.vela@wsu.edu; Associate Dean of University Libraries, Director of the Health Sciences Library, and Director, National Network of Libraries of Medicine Pacific Northwest Region, University of Washington, Seattle, WA, bardyn@uw.edu

## Abstract

**Background:**

HEALWA is an online library of evidence-based health information resources that are available to Washington state health practitioners. To increase awareness and use of HEALWA among health practitioners in rural areas, the National Network of Libraries of Medicine Pacific Northwest Region and Washington State University Spokane co-funded an outreach librarian position to provide instruction on using HEALWA.

**Case Presentation:**

After attempts at frequent in-person workshops failed due to lack of attendance, a one-hour-long webinar targeted at rural nurses was developed to be delivered once a month. These webinars introduced participating health professionals to HEALWA, including how to set up their access and how to navigate the resource. To accommodate the busy schedules and different learning styles of the target audience, the workshops occurred both as monthly webinars and in-person, when available, in addition to an online self-guided tutorial. Continuing education credit was obtained through the Washington State Nurses Association, and a partnership with the Washington State Nursing Commission improved promotion of the webinars. Evaluations for both the webinars and workshops have been largely positive.

**Conclusions:**

The webinar series, coupled with in-person workshops and an online tutorial, reached nurses in rural areas of Washington state to increase awareness of HEALWA. To further facilitate access to HEALWA instruction, a recorded version of the live webinar is in development.

## BACKGROUND

HEALWA is an online portal for reliable health information, known as the “on-call library for Washington state practitioners.” A 2007 state bill mandated the creation of HEALWA, stating that “health professionals require easy and timely access to medical journals and other related scientific and evidence-based information on a regular basis to enhance evidence-based medical practice” [1]. The online access to resources referenced in the bill was to be hosted and managed by the Health Sciences Library at the University of Washington in partnership with the Washington State Department of Health.

After two years of development, HEALWA was launched in January 2009. During the time that it has been in operation, HEALWA has provided access to reliable health information resources including over 50 databases covering a wide range of topics such as diagnosis, drugs and medications, and patient information; more than 2,000 electronic books covering general medical topics and profession-specific areas; and journals with full-text article access. Participating health professions have access to HEALWA stemming from a small fee that is included with their licensing fees. As of 2017, the number of participating health professions has grown from the original 14 professions listed in the 2007 bill to 24 professions as more health professions realize the value of access to information (supplemental Appendix A).

A small number of existing programs are similar to HEALWA. Some of these electronic health libraries (EHLs) provide access to health information to affiliated members of a consortium and are funded through membership fees from participating institutions. Examples of these programs include the Arizona Health Information Network and the Hospitals and University at Buffalo Library Resource Network [2, 3]. Other EHLs provide access to health information to citizens of their state, like GALILEO in Georgia [4]. HEALWA is unique among these existing programs because it is funded through state-collected fees and provides access to eligible health practitioners from any hospital or clinic in Washington state.

Some larger hospital systems in the state provide resources like UpToDate to their providers, but these resources can be cost-prohibitive for smaller hospital systems and individual clinics. The primary benefit of HEALWA is that it ensures equity of access to health information, regardless of the resources that individual hospitals or clinics provide. This is important in Washington because most of the state is classified as rural (defined as counties with a population density less than 100 people per square mile or counties smaller than 225 square miles), with 16% of the state’s population living in rural counties [5–7]. Additionally, 6% of registered nurses (RNs) and 8% of licensed practical nurses (LPNs) in Washington practice in rural areas, compared to 15% of RNs and 24% of LPNs nationally [8–10]. Access to HEALWA and the resources it provides are invaluable to health practitioners at small, rural clinics and hospitals that would not otherwise have access to these resources.

An evaluation study was conducted in 2015 to determine the impact of HEALWA on patient care and the value of HEALWA to health practitioners in the state. The survey results indicated that 78% of eligible users had not used HEALWA, mostly due to lack of awareness of its existence. However, the 22% of eligible users who had used HEALWA reported that it contributed to higher quality of care, provided clinical value, resulted in better informed clinical decisions, and provided them with new knowledge. Based on the survey, it was recommended that an outreach approach could improve awareness of HEALWA throughout Washington, especially in rural areas.

There are some unique challenges to conducting outreach to rural nurses. They are often geographically isolated, making access to peers, libraries, and continuing education opportunities challenging [11–14]. Rural clinics and hospitals often have limited financial resources, making the cost of travel and educational opportunities another barrier to outreach [12–14]. While Internet connectivity has vastly improved in rural areas, nurses still face a shortage of computer stations and often feel they lack the skills necessary to use them effectively [14]. Lack of time to search for health information and inadequate information literacy are frequently cited challenges for all health practitioners [15].

Online instruction is a possible solution to the barriers caused by in-person offerings. While in-person, lecture-style learning is often the preferred mode of instruction for nurses, there is a willingness to use other instructional delivery methods, including webinars and self-study [11, 16]. Online instruction offers flexibility that is not offered by in-person instruction, as the learning can take place from any location with an Internet connection, rather than in a single physical location [17]. This flexibility saves travel costs and limits the need to take time away from work. However, computer literacy can be a potential issue for the effectiveness of online instruction; technology skills are a prerequisite for a successful online learning experience, and many nurses lack confidence in this area [17].

## STUDY PURPOSE

To promote the use of HEALWA and improve the information discovery skills of health practitioners in rural eastern Washington state, the newly formed Elson S. Floyd College of Medicine (ESFCOM) at Washington State University (WSU) Spokane and the National Network of Libraries of Medicine (NNLM) Pacific Northwest Region (PNR) partnered to fund a new health sciences outreach librarian position at WSU Spokane. The position was filled in August 2016 and is expected to be funded through 2021. This outreach librarian has worked with the HEALWA support team at the UW Health Sciences Library to increase the awareness and usage of HEALWA among rural nurses in that region using a variety of outreach strategies, the most successful of which has been a regularly occurring online instructional webinar. This report describes the effort to provide outreach education about HEALWA to rural nurses in eastern Washington state through online webinars.

## CASE PRESENTATION

As there are approximately 196,850 eligible HEALWA users, the authors chose to focus early outreach efforts on the largest eligible health profession group: nurses (RNs and LPNs). When this project began in 2016, this population represented 58% of eligible HEALWA users [18]. Additionally, there were a number of large, active professional associations for nurses in Washington state that could facilitate the development of a network of contacts for outreach.

To educate this specific population about using HEALWA, we developed a one-hour-long workshop to teach participants how to access and navigate the HEALWA website. The workshop was originally planned to be delivered through a face-to-face instructional format at three locations throughout eastern Washington in December 2016. However, a lack of registrations from health practitioners in those communities led us to cancel these workshops. This setback prompted us to explore the possibility of delivering the HEALWA workshop content via an online webinar, which would eliminate the need for busy health practitioners to physically travel to a location and provided the opportunity for recording sessions for later viewing by those who could not attend.

We converted the in-person workshop to an online format using Microsoft PowerPoint, and we purchased Adobe Connect to facilitate its online delivery. After re-promoting the workshops as webinars via emails sent to health districts and hospitals in the region, we held 2 sessions in January 2017 with a total of 11 participants. Feedback was largely positive from the 8 participants who completed post-webinar evaluations, with all respondents indicating that they had learned something new (100%) and that they intended to use HEALWA in the future (100%).

**Figure 1:**
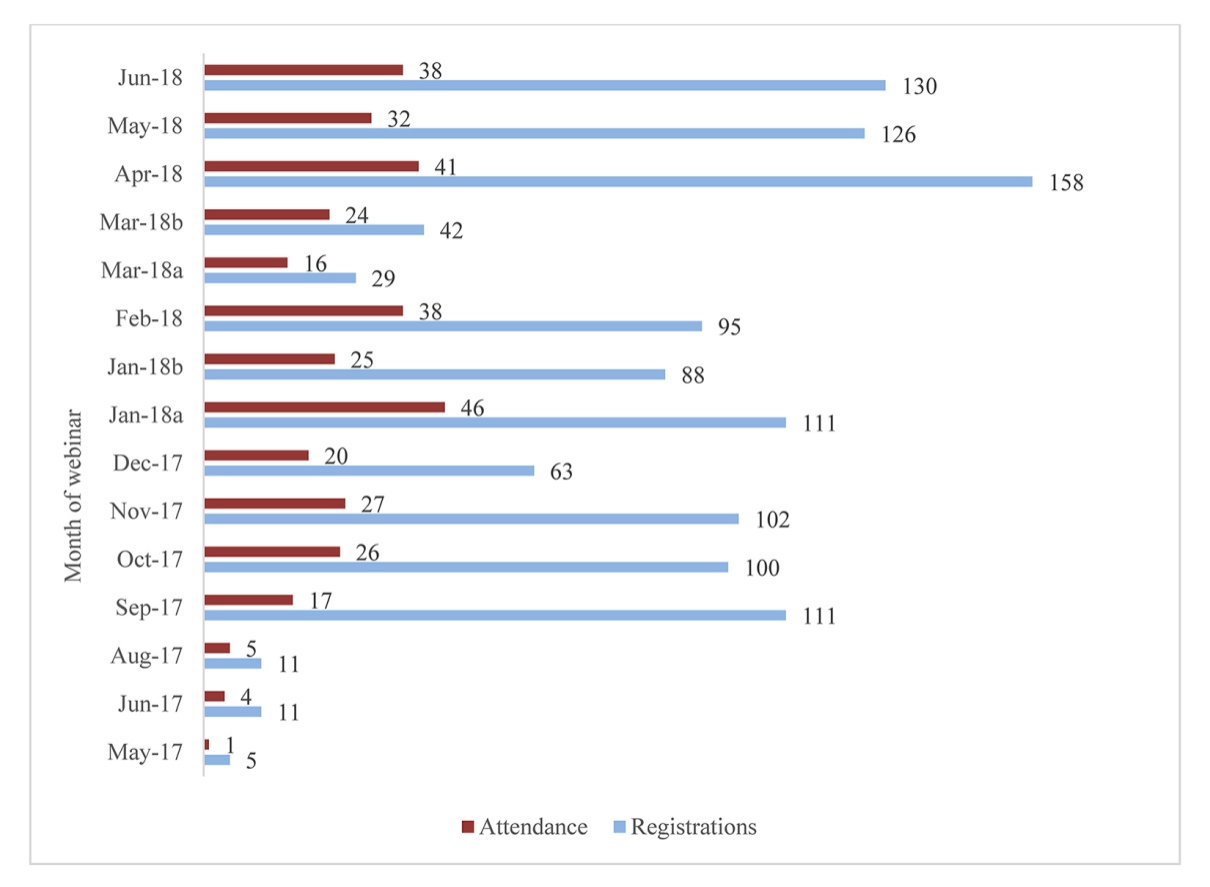
Number of registrations and attendees per webinar

As a result of this positive feedback regarding the webinar delivery of HEALWA instruction, we decided to provide a regular schedule of once-per-month webinars, each of which would deliver the same introductory content that would give attendees the information that they needed to begin using HEALWA effectively. To further encourage attendance, we collaborated with the WSU College of Nursing to obtain accreditation from the Washington State Nurses Association (WSNA) for 1 contact hour of continuing nursing education (CNE) for the webinar, which was received in April 2017. This accreditation provided nurses with an additional incentive to attend the webinars. As of June 2018, there have been 15 workshops via webinar, with a total of 360 attendees (Figure 1).

### Promotion and scheduling

In the early stages of the monthly webinar offerings, we promoted each webinar via email through a growing network of contacts that included county health officials, hospital education staff, and state and regional health associations. Emails were sent to these contacts 1 month prior to each webinar. This approach was not effective in increasing attendance, as shown by the low attendance numbers from May to August 2017. In September 2017, we partnered with the Washington State Nursing Commission to begin promoting the webinars through their biannual newsletter, which is distributed to more than 100,000 licensed and student nurses throughout the state of Washington. This promotion method was more successful in increasing attendance, as shown by the large increase in registrations and attendance between September 2017 and April 2018.

For the first 8 months of the webinar series, the webinars were held on the first Tuesday of each month at 5:00 p.m., Pacific time. This was due to our availability and assumptions that most nurses would be available to attend a webinar in the evening. In December 2017, to explore the possibility that a different webinar schedule could improve attendance, we distributed a 2-question survey through the Washington State Nursing Commission that asked participants about the best day of the week and time of day to attend a 1-hour webinar. Based on the over 2,000 responses collected, the timing of the webinar was changed to 10:00 a.m., Pacific standard time, in January 2018, and subsequent sessions saw an increase in registrations and attendance.

### Webinar evaluations

After each webinar, attendees were asked to complete a short 3-question evaluation survey in order to receive their CNE certificate (supplemental Appendix B). This survey was based on an evaluation survey from the NNLM Training Office (NTO). The survey was created in Qualtrics and distributed via anonymous link both after the webinar and in a follow-up email that was sent to attendees the day after a webinar. Out of a total of 364 attendees of the 15 webinars, 359 evaluations were collected, giving a response rate of 99%; however, this should likely be lower given that there were multiple instances of groups of people attending the webinar under a single name and people completing the evaluation survey twice. The combined results of the evaluation surveys were largely positive, with nearly all respondents indicating that they felt they had an improved ability to find health information online (n=324, 90%), that they planned to start using HEALWA (n=351, 98%), and that they planned to tell their peers about HEALWA (n=348, 97%).

A similar evaluation survey was distributed to webinar attendees approximately 1 month after they attended a webinar to examine the effect of the outreach instruction after attendees had time to further explore and use HEALWA (supplemental Appendix C). A total of 116 responses were collected out of 351 follow-up evaluation surveys that were distributed for 12 webinars, giving a response rate of 33%. Of these responses, 71% reported that they recalled the webinar very or extremely well (n=82). Three-quarters of respondents indicated that they used HEALWA after they attended the webinar (n=89, 77%), and two-thirds of respondents indicated that they felt the webinar improved their ability to find health information online (n=75, 65%).

### In-person workshops

While most HEALWA education and outreach took place online, we also presented the workshop to interested groups in a face-to-face format when schedules could be coordinated. A total of 9 workshops have been delivered to groups that included school nurses, nutritionists, and physicians at educational service districts, public health departments, and hospital teams. Attendance at these workshops averaged 11 participants, and the immediate feedback from paper evaluations forms has been positive, with 100% of respondents (n=74) indicating that they learned about a new resource and a new skill that they planned to use in the future. Additionally, 93% of respondents (n=69) reported that the webinar improved their ability to find useful health information online.

### Online tutorial

To support all learning preferences and to consider the busy schedules of health practitioners, we created a self-guided HEALWA tutorial using the LibGuides platform [19]. The tutorial was created for all health practitioners and was promoted during webinars and workshops. The tutorial consists of a series of short instructional videos that we produced using Camtasia along with printable guides created using Microsoft PowerPoint. There are also quizzes available to users who would like to test their knowledge of how to use HEALWA, which were created using Google Forms. The tutorial was launched in October 2016 and promoted at the end of each webinar. As of June 2018, the tutorial had 2,579 views.

## DISCUSSION

Providing introductory HEALWA instruction online has given us the opportunity to reach a larger number of health practitioners in Washington state than would have been possible through in-person workshops. The online format accommodates the busy and variable schedules of health practitioners, while allowing live demonstrations and conversations around using HEALWA to locate health information. Offering in-person workshops on an ad hoc basis along with the online self-guided tutorial provides multiple avenues for health practitioners to engage with HEALWA instruction.

### Challenges

The greatest challenge to the HEALWA webinars has been the large percentage of registrants who fail to attend the sessionswhich allowed the outreach librarian. On average, 70% of people who registered for a HEALWA webinar were no-shows. This is standard for webinar offerings, as an average of 36% of webinar registrants actually attend live sessions [20]. The webinar no-shows experienced in this outreach project were most likely due to busy and changing schedules, something common to all health care practitioners. Another possible reason for the no-shows was that people simply forgot that they had registered to attend the webinar. While there is little that can be done to address the former reason, the latter could be alleviated with reminder emails prior to the webinar. For example, the BrightTALK 2017 Webinar Benchmarks Report recommends promoting webinars beginning 3–4 weeks prior to the scheduled webinar and continuing up to 15 minutes prior to the webinar itself [20].

Computer literacy is a common challenge for health practitioners [14], and a small number of practitioners who wanted to attend a webinar were not familiar with the technical aspects of joining an online meeting space like Adobe Connect. Some requests for troubleshooting were made prior to webinar sessions, which allowed us to provide some additional instructions. However, most requests for assistance were made during the webinars, when we were unable to respond. It was possible that some practitioners had technical difficulties, did not request troubleshooting assistance, and failed to attend the webinar, which would also contribute to the attendance issue described above. This problem has been addressed with the presence of another person to act as a technical assistant during the webinars, but there have been some scheduling conflicts that makes this type of assistance inconsistent. Other continuing education webinar programs have recommended providing registrants with training in computer skills or simple text instructions for accessing the webinar [21, 22].

### Future directions

Both evaluation survey comments from webinar attendees and informal feedback from other practitioners who were interested in learning about HEALWA prompted us to consider creating a recorded version of the webinar. A recorded version of the webinar would provide more flexibility in viewing opportunities for those who want to learn more about using HEALWA: they would be able to watch the webinar whenever they had time rather than being limited to the once-per-month live sessions. It would also be a more efficient means for us to reach a larger number of rural nurses, in terms of cost, time, and effort. WSNA and the Washington State Nursing Commission would be able to promote the webinar on their websites and through their communications with members. Details about how this webinar recording would be evaluated are still being determined.

We plan to expand the reach of this project by targeting other eligible health professions groups. The same webinar, workshop, and online tutorial approaches that were used to conduct outreach to rural nurses could be used to increase awareness among other groups. There are other health professions associations like WSNA that could be partners in reaching these other groups; for example, the Washington Occupational Therapy Association has expressed an interest in a webinar offering like the one provided to nurses. The challenges that arose while providing outreach to rural nurses would still be present, in other words, no-shows and technical difficulties; however, we believe that providing additional technical support and webinar reminders would help alleviate these issues. If the recorded version of the webinar for rural nurses is successful, then providing a recorded webinar tailored to other health professions could be a solution to these challenges. Additionally, portions of the instructional content would need to be changed to address the specific information needs of these other health professions.

Through these activities, we will continue to work to increase the number of HEALWA users in eastern Washington and improve access to reliable health information for health practitioners in this region. Such access will ultimately serve to enhance patient care and positive patient outcomes. By using multiple approaches, we may be able to overcome the typical barriers to rural health practitioner outreach and ensure that this population has every opportunity to learn more about this valuable resource.

## SUPPLEMENTAL FILES

Appendix A Health professions that are eligible for HEALWA access

Appendix B Post-webinar evaluation survey

Appendix C One-month follow-up evaluation survey
