# Supplementary material for: Increasing rural nurses’ awareness of a statewide health information resource: an educational outreach initiative
Source: J Med Libr Assoc. 2019 Apr 1;107(2):244–50. doi: 10.5195/jmla.2019.542 (PMC6466490; doi:10.5195/jmla.2019.542)
Supplement: Appendix A [file jmla-107-244-s001.pdf]

JMLA Journal of the Medical Library Association

SC
Supplemental content to
J Med Libr Assoc. Apr;107(2):dx.doi.org/10.5195/jmla.2019.542
www.jmla.mlanet.org
© Vela, Bardyn 2019

Increasing rural nurses' awareness of a statewide health information resource: an educational outreach initiative

Kathryn Vela, MLIS, AHIP; Tania Bardyn, MLIS, AHIP

APPENDIX A

Health professions that are eligible for HEALWA access

Chiropractors
Clinical social work associates
Dietitians
East Asian medicine practitioners
Licensed marriage and family therapists
Licensed practical nurses
Marriage and family therapy associates
Massage therapists
Mental health counselors
Midwives
Naturopathic physicians
Nutritionists
Occupational therapists
Occupational therapy assistants
Optometrists
Osteopathic physician assistants
Osteopathic physicians
Physician assistants
Physicians
Podiatrists
Psychologists
Registered nurses
Social workers
Speech language pathologists

heal-wa.org/about/healwa-eligible-professions/
