# Supplementary material for: Increasing rural nurses’ awareness of a statewide health information resource: an educational outreach initiative
Source: J Med Libr Assoc. 2019 Apr 1;107(2):244–50. doi: 10.5195/jmla.2019.542 (PMC6466490; doi:10.5195/jmla.2019.542)
Supplement: Appendix B [file jmla-107-244-s002.pdf]

JMLA Journal of the Medical Library Association

SC
Supplemental content to
J Med Libr Assoc. Apr;107(2):dx.doi.org/10.5195/jmla.2019.542
www.jmla.mlanet.org
© Vela, Bardyn 2019

Increasing rural nurses' awareness of a statewide health information resource: an educational outreach initiative

Kathryn Vela, MLIS, AHIP; Tania Bardyn, MLIS, AHIP

APPENDIX B

Post-webinar evaluation survey

Title of training session: Using HEALWA: A Health Evidence Resource for Washington State

Organization:

Date: [Month, Day, Year]

Your feedback will allow the National Network of Libraries of Medicine (NNLM) to assess and improve its training program for all participants. Your responses to this form are anonymous. Summaries of course feedback may be reported publicly, but your specific responses will not be reported. Your participation is voluntary, but we value your assessment and hope you will provide it.

1. Did this training session introduce you to at least one health information resource or tool that you had never used before?

o Yes
o No
o Not applicable

2. Did you learn a new skill in this training session that you plan to use in the future?

o Yes
o No
o Not applicable

3. Please indicate your level of agreement with each statement:

	Strongly agree	Somewhat agree	Somewhat disagree	Strongly disagree	Not applicable
This training improved my ability to find useful online health information.	O	O	O	O	O
I plan to start using at least one resource or tool that I learned about in this training.	O	O	O	O	O
I plan to tell others about at least one resource or tool that I learned about in this training.	O	O	O	O	O
