# Supplementary material for: Increasing rural nurses’ awareness of a statewide health information resource: an educational outreach initiative
Source: J Med Libr Assoc. 2019 Apr 1;107(2):244–50. doi: 10.5195/jmla.2019.542 (PMC6466490; doi:10.5195/jmla.2019.542)
Supplement: Appendix C [file jmla-107-244-s003.pdf]

JMLA Journal of the Medical Library Association

SC

Supplemental content to
J Med Libr Assoc. Apr;107(2):dx.doi.org/10.5195/jmla.2019.542
www.jmla.mlanet.org
© Vela, Bardyn 2019

Increasing rural nurses' awareness of a statewide health information resource: an educational outreach initiative

Kathryn Vela, MLIS, AHIP; Tania Bardyn, MLIS, AHIP

APPENDIX C

One-month follow-up evaluation survey

Title of training session: Using HEALWA: A Health Evidence Resource for Washington State

Organization:

Date: [Month, Day, Year]

Your feedback will allow the National Network of Libraries of Medicine (NNLM) to assess and improve its training program for all participants. Your responses to this form are anonymous. Summaries of course feedback may be reported publicly, but your specific responses will not be reported. Your participation is voluntary, but we value your assessment and hope you will provide it.

1. How well do you recall the workshop you attended approximately one month ago?
a. Extremely well
b. Very well
c. Moderately well
d. Slightly well
e. Not well at all

2. Do you use the resources you learned about in that workshop?
a. Yes
b. No
c. I'm not sure

3. Please indicate your level of agreement with each statement:

	Strongly agree	Somewhat agree	Somewhat disagree	Strongly disagree	Not applicable
The training improved my ability to find useful online health information.	O	O	O	O	O
I have used at least one resource or tool that I learned about in this training.	O	O	O	O	O
I have told others about at least one resource or tool that I learned about in this training.	O	O	O	O	O

JMLA Journal of the Medical Library Association
SC
Supplemental content to
J Med Libr Assoc. Apr;107(2):dx.doi.org/10.5195/jmla.2019.542
www.jmla.mlanet.org
© Vela, Bardyn 2019

4. Would you recommend this workshop to someone else?

a. Yes
b. Maybe
c. No
